# Loss of Smell and Taste in Patients With Suspected COVID-19: Analyses of Patients’ Reports on Social Media

**DOI:** 10.2196/26459

**Published:** 2021-04-22

**Authors:** Sachiko Koyama, Rumi Ueha, Kenji Kondo

**Affiliations:** 1 Department of Chemistry Indiana University Bloomington, IN United States; 2 Department of Otolaryngology and Head and Neck Surgery Faculty of Medicine The University of Tokyo Tokyo Japan

**Keywords:** COVID-19, anosmia, ageusia, free reports on social media, symptomatic, asymptomatic, recovery of senses, symptom, social media, smell, taste, senses, patient-reported, benefit, limit, diagnosis

## Abstract

**Background:**

The year 2020 was the year of the global COVID-19 pandemic. The severity of the situation has become so substantial that many or even most of the patients with mild to moderate symptoms had to self-isolate without specific medical treatments or even without being tested for COVID-19. Many patients joined internet membership groups to exchange information and support each other.

**Objective:**

Our goal is to determine the benefits and limits of using social media to understand the symptoms of patients with suspected COVID-19 with mild to moderate symptoms and, in particular, their symptoms of anosmia (loss of the sense of smell) and ageusia (loss of the sense of taste). The voluntary reports on an internet website of a membership group will be the platform of the analyses.

**Methods:**

Posts and comments of members of an internet group known as COVID-19 Smell and Taste Loss, founded on March 24, 2020, to support patients with suspected COVID-19 were collected and analyzed daily. Demographic data were collected using the software mechanism called Group Insights on the membership group website.

**Results:**

Membership groups on social media have become rare sources of support for patients with suspected COVID-19 with mild to moderate symptoms. These groups provided mental support to their members and became resources for information on COVID-19 tests and medicines or supplements. However, the membership was voluntary, and often the members leave without notification. It is hard to be precise from the free voluntary reports. The number of women in the group (6995/9227, 75.38% as of October 12, 2020) was about three times more than men (2272/9227, 24.62% as of October 12, 2020), and the peak age of members was between 20-40 years in both men and women. Patients who were asymptomatic other than the senses comprised 14.93% (53/355) of the total patients. Recovery of the senses was higher in the patients who were asymptomatic besides having anosmia and ageusia. Most (112/123, 91.06%) patients experienced other symptoms first and then lost their senses, on average, 4.2 days later. Patients without other symptoms tended to recover earlier (*P*=.02). Patients with anosmia and ageusia occasionally reported distorted smell and taste (parosmia and dysgeusia) as well as experiencing or perceiving the smell and taste without the sources of the smell or taste (phantosmia and phantogeusia).

**Conclusions:**

Our analysis of the social media database of suspected COVID-19 patients’ voices demonstrated that, although accurate diagnosis of patients is not always obtained with social media–based analyses, it may be a useful tool to collect a large amount of data on symptoms and the clinical course of worldwide rapidly growing infectious diseases.

## Introduction

Recent outbreaks of COVID-19, caused by SARS-CoV-2, have escalated into a worldwide pandemic. Over 29.4 million people in the United States alone have contracted the virus, causing over 532,000 deaths as of March 13, 2021.

According to the World Health Organization (WHO), “serious symptoms” are defined as “difficulties in breathing or shortness of breath, chest pain or pressure, loss of speech or movement” [1]. The Centers for Disease Control and Prevention (CDC) reported that over 80% of patients with COVID-19 have mild to moderate levels of illness [2] and 20% of the patients develop severe to critical conditions. Although there have been a number of studies published regarding the symptoms and their clinical course for COVID-19, most of them have focused on the patients with severe symptoms, and there have been few reports on patients with COVID-19 with mild to moderate conditions. Depending on the country, especially where the numbers of cases have been high and beds at hospitals are limited, most of the patients with mild to moderate symptoms of suspected COVID-19 have been self-isolating themselves with minimum access to medical treatments due to the large number of patients with severe conditions. Previously there were criteria that were required to be present (fever and coughs) to get tested, although later it became clearer that the symptoms vary largely and that made many suspected patients with various symptoms not be allowed to get tested. The patients with COVID-19 with mild to moderate symptoms are thus the most invisible population of patients who have received the least attention from the public and from medical care facilities.

Recently it has been shown that there is a high percentage (40% to as high as 96%) of asymptomatic [3-6] or presymptomatic [7] COVID-19 positive patients. In addition, more recently, there have been reports on the loss of the senses of smell (anosmia) and taste (ageusia) in COVID-19 positive patients with otherwise asymptomatic to mild levels of other symptoms [8-11] and as one of the early stage symptoms [9]. The ability to sense smell and taste showed a correlation with the severity of other symptoms as well, and odors related to chemesthesis were also found to be impaired by contraction of SARS-CoV-2 [12,13]. Studies have shown that 98% of patients with COVID-19 showed some smell dysfunction [14], indicating that smell dysfunction is a major biomarker of COVID-19. In a survey comparing COVID-19 positive and negative participants, smell dysfunction was found to be the best predictor of COVID-19 [15,16]. There are also papers reporting that asymptomatic patients with COVID-19 stay contagious longer than patients with symptoms [17]. These studies suggest that there is an urgent need to understand the asymptomatic to mild and moderate symptoms of patients with COVID-19 with anosmia and ageusia. Asymptomatic conditions sometimes mean that the patients actually have symptoms but did not realize or notice them because they are mild or because these symptoms were not included in the list of well-known symptoms like coughs and fevers. Understanding of the symptoms and the progression of COVID-19, especially in the mild cases of COVID-19, may help the subjectively asymptomatic patients notice any subtle symptoms that others have previously ignored. This includes the symptoms of anosmia and ageusia. The understanding of the onset and progression of anosmia and ageusia in otherwise asymptomatic patients and that of patients with mild or moderate symptoms may contribute to containment of the virus by enabling the detection of patients with COVID-19 at an earlier stage after contracting SARS-CoV-2.

Near the start of the pandemic, a group to support patients who lost their senses of olfaction or taste due to contraction of COVID-19, known as *COVID-19 Smell and Taste Loss*, was founded on a social media site. The posts from the members were recorded at that time to provide *precision advice*, which was based on the concept of the *Precision Medicine Initiative*. *Precision Medicine* is an approach for disease treatment that considers individual variability. In the case of a social media group, one of the ways to provide *precision advice* is to take into consideration the history of each person’s previous posts on their symptoms and progress. The record of the posts at the early stage after the group was founded was conducted with the purpose of understanding the symptoms of the new disease in each person, which became the base of this study. To understand the new disease, COVID-19, we recorded all the posts on symptoms whether they were related to the senses or not.

The aim of this study was to analyze the posts and comments on this social media membership group to establish the benefits and limits of using social media to understand COVID-19 and to investigate the symptoms of mild to moderate COVID-19 patients, especially focusing on anosmia and ageusia. As earlier studies reported, these symptoms are highly observed in patients with COVID-19 [14]. They are early signs of COVID-19 [9], and in some SARS-CoV-2 infected patients, they were the only symptoms of COVID-19 [9,11]. There are only a few papers so far using the reports of patients obtained from social media [18,19]. This is one of the few reports on the mildly to moderately affected patients, many of whom did not have enough symptoms to receive COVID-19 tests and medical treatment, and therefore, clinical information is limited.

## Methods

### Recording of Cases

A membership group called *COVID-19 Smell and Taste Loss* was established on March 24, 2020, by the charity organization AbScent, which is based in England, United Kingdom. Polls were occasionally conducted to determine various aspects of the members, for example, hospitalization for COVID-19. The information on the age, sex, and the countries and cities where the members were located was obtained through the software mechanism called *Group Insights*. The reports posted were recorded daily as they were in Excel (Microsoft Corporation) spreadsheets with the records of the dates posted and the names, COVID-19 test results if available, a short summary of the symptoms, starting dates of anosmia and ageusia symptoms if available, and recovery dates from these symptoms if available. When the same person posted on a separate day, the posts were recorded under the name of the person with the records of the dates they were posted. Recording of the posts and comments for this study lasted from March 24 to July 1, 2020. Posts and comments without specific content related to their own symptoms were not recorded (eg, expressing their sympathy, excitement, or approval saying “Yes,” “congratulations!” “Thank you,” or “nobody knows”).

### Institutional Review Board

The study is approved as exempt (Institutional Review Board protocol number 10082020-1, Exempt Category #3)

### Case Studies

Because, in this group, members freely reported their cases, the level of detail for each member was not consistent. We classified the reports by whether the patient had other symptoms than anosmia and ageusia, and whether they recovered their senses, and then selected several reports that described their symptoms in detail.

### Statistical Analysis

Fisher exact test was used to statistically evaluate the recovery rate among groups.

## Results

### Demographic Data

**Figure 1:**
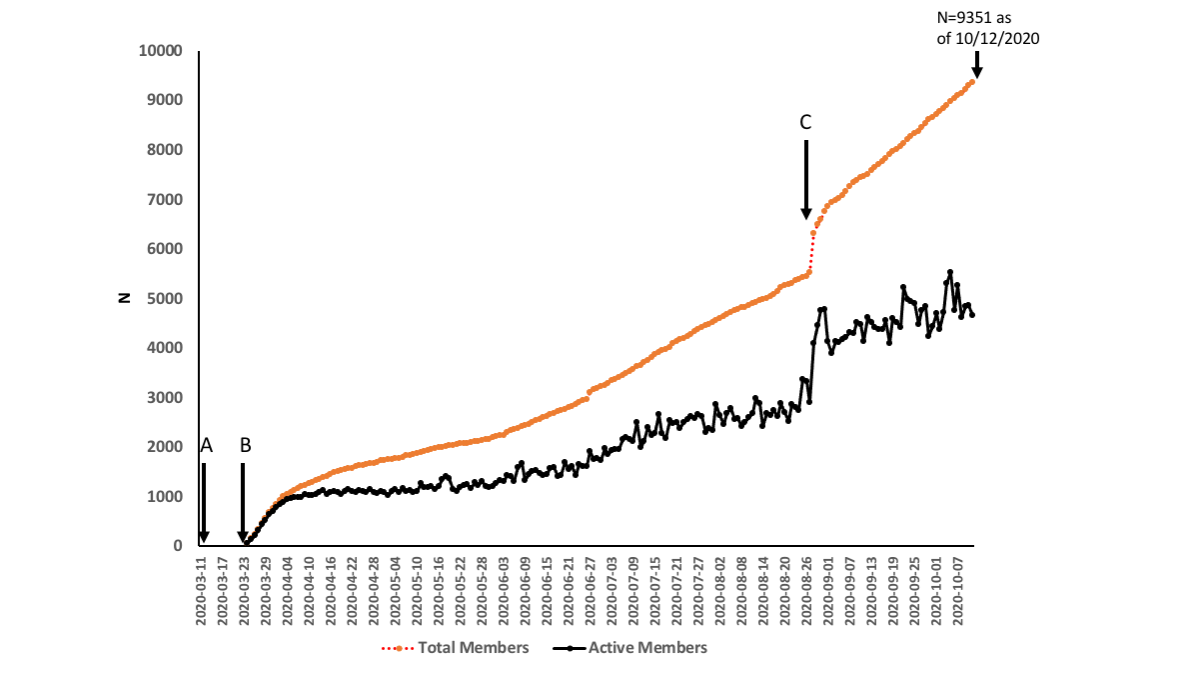
Increase in the number of members (red) and active members who make posts or comments to other posts (black). A: Date of the World Health Organization announcement on the pandemic; B: COVID-19 Smell and Taste Loss membership group founded; C: increase of over 1000 members within a few days following the interview of the founder of the group on the BBC.

Figure 1 shows the time course since the pandemic started. On March 11, 2020, the WHO declared the rapidly spreading SARS-CoV-2 virus a pandemic and warned that it might spread to many countries around the world (Figure 1A). The disease caused by the virus, COVID-19, was first considered to cause fever and coughs as the main symptoms. However, soon it was discovered that there were more symptoms, including the loss of the senses of smell (anosmia) and taste (ageusia). The membership group on social media studied in this project was founded on March 24 (Figure 1B), and the number of members reached about 1000 during the first week (Figure 1). This immediate increase of the members indicates the rapid spread of the disease and the large number of people who suddenly lost their senses of smell and taste at the early stage of the pandemic. The increase in the number of members showed another surge around the time when the second wave in the number of cases started in the autumn of 2020. This may be due to both the increased publicity after the introduction of the group in the media (Figure 1C) and the large number of people with COVID-19–induced anosmia and ageusia. This suggests the existence of a large number of patients who lost their senses during this pandemic.

**Figure 2:**
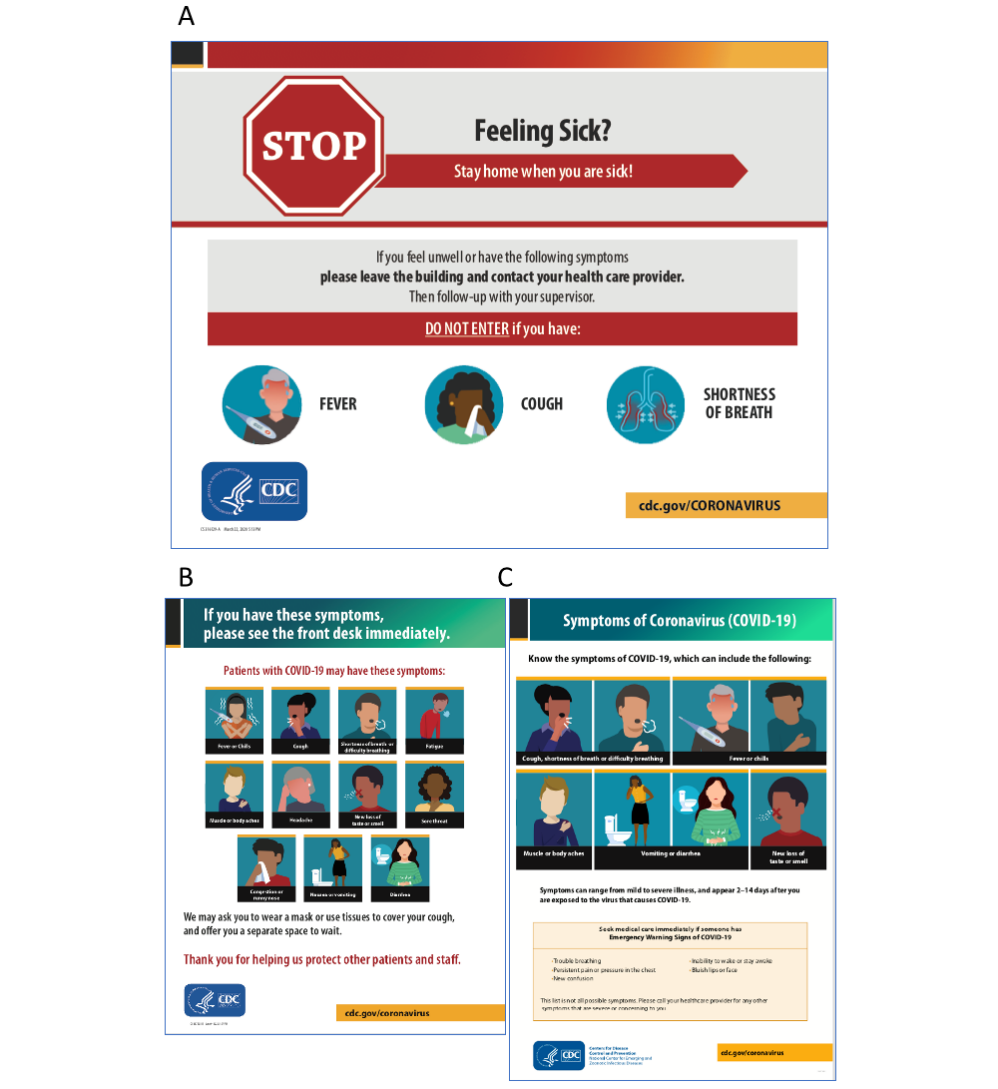
Indoor and outdoor signs released by the CDC as free to be printed and used. CDC: Centers for Disease Control and Prevention.

**Figure 3:**
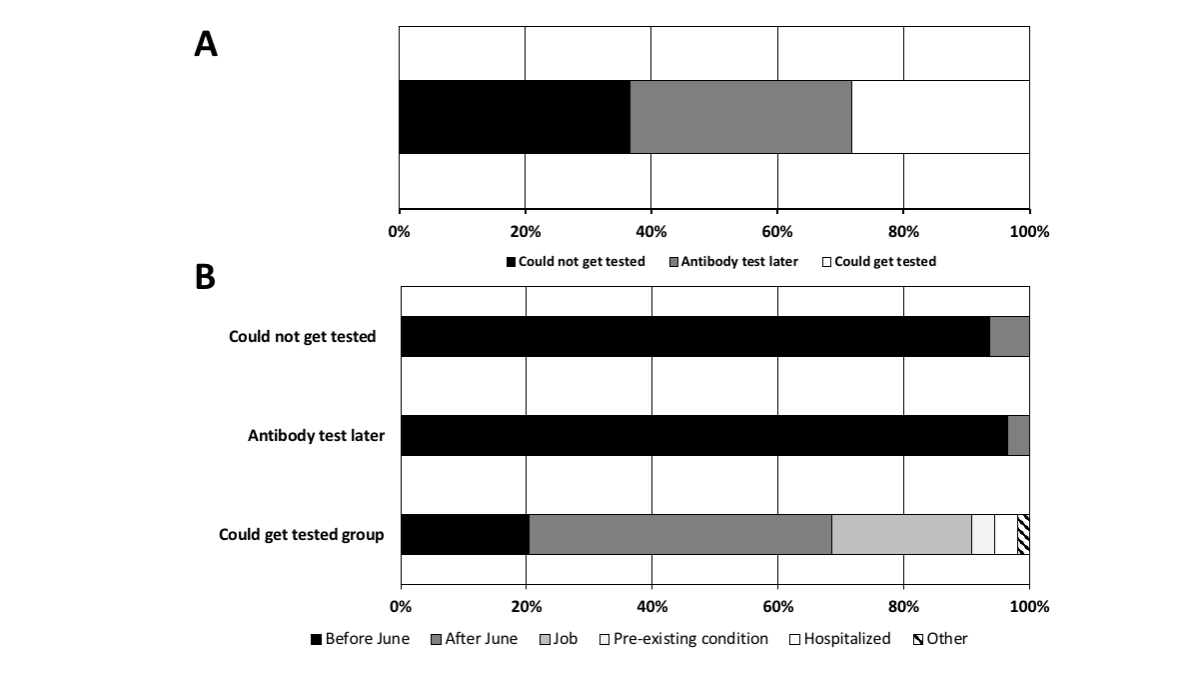
Summary of the reply to the question “were you able to get tested immediately after the onset of the symptoms of COVID-19” (A) and classification of the comments related to their selection. (B) Most often listed reasons that they could or could not get tested were the time of onset (before June or after June when the Centers for Disease Control and Prevention announced changes in the typical symptoms and included more symptoms), their jobs as essential workers or supervisors’ arrangements, pre-existing health conditions, and hospitalization because of symptoms.

What were considered the symptoms of COVID-19 have changed from the earlier stage of the pandemic in spring 2020 to summer and fall 2020. Figure 2 shows the outdoor and indoor signs made by the CDC in March, June, and July [20]. In the sign generated in March (Figure 2A), only the symptoms of fever, coughs, and shortness of breath are described, whereas in the June and July signs, the variety of symptoms has increased by more than double, and now it is well known that not every COVID-19 positive patient has fever, coughs, or shortness of breath (Figure 2B and C). In the poll asking if they got the COVID-19 tests immediately after the onset of symptoms, about 70% (n=259) of the 705 people answered that they could not get tested at all or could not get tested immediately and received the antibody tests later (Figure 3A; could not get tested: n=259, 36.74%; antibody test later: n=247, 35.04%). Analyses of the people who added comments (n=54) to the poll revealed that most of the patients with COVID-19 who could get tested immediately after the onset of symptoms was due to their jobs (essential workers, mostly) or those who contracted the virus after June when the criteria to get tested expanded (compare Figure 2A and 2B; Figure 3B bottom; job: n=12, 22.22%; after June: n=26, 48.15%). The comments by the people who replied that they could not get tested immediately and received antibody tests later or could not get any test revealed that they were mostly the people who had an onset of symptoms before June (Figure 3B, top two bars).

**Figure 4:**
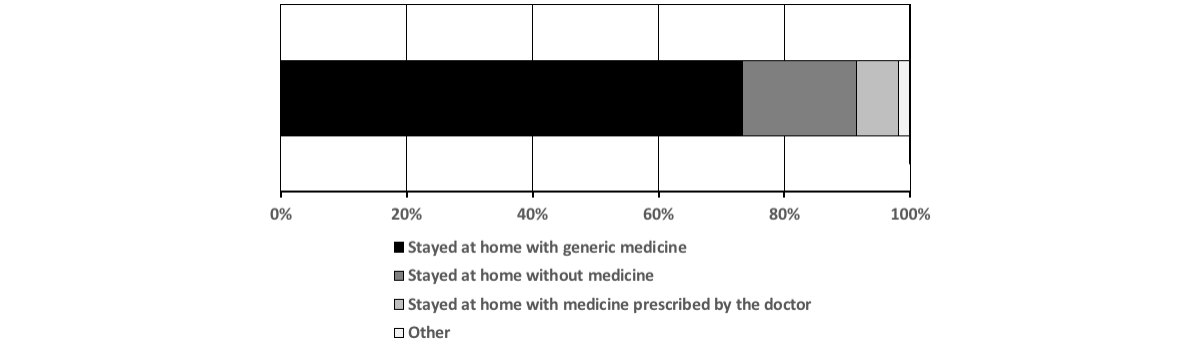
Availability of medical treatments. The result of another choice "hospitalized and then stayed at home with medicines prescribed by the doctors" is not shown as no one selected the option.

Thus during the early stage of the pandemic, there were many people who got sick and self-diagnosed themselves with COVID-19 (suspected COVID-19 patient). This inability to get a COVID-19 test immediately after the onset of symptoms also resulted in the inability to get immediate medical treatments because medicines were not prescribed without an official diagnosis. We asked a question about the way they spent time after the onset of symptoms and found that about 90% (n=204,) of the 221 members who replied either stayed at home with generic medicines and supplements (n=162, 73.73%) or stayed at home without any medicines (n=40, 18.10%; Figure 4). Only 6.79% (n=15) replied that they stayed at home with medicines prescribed by doctors. Although there were some responses showing that there were members who experienced hospitalization in a separate poll (see Figure 3), none of them responded to the poll on the medical treatments, and thus, the option of medical treatment following hospitalization is not shown in Figure 4. Although there is some missing information on the hospitalized people in the results, Figure 4 clearly shows the invisible nature of the patients with mild to moderate symptoms, which also suggests that the number of cases worldwide in the countries where availability of COVID-19 tests was limited or had criteria to get tested is highly likely to be far more than the officially reported numbers.

**Figure 5:**
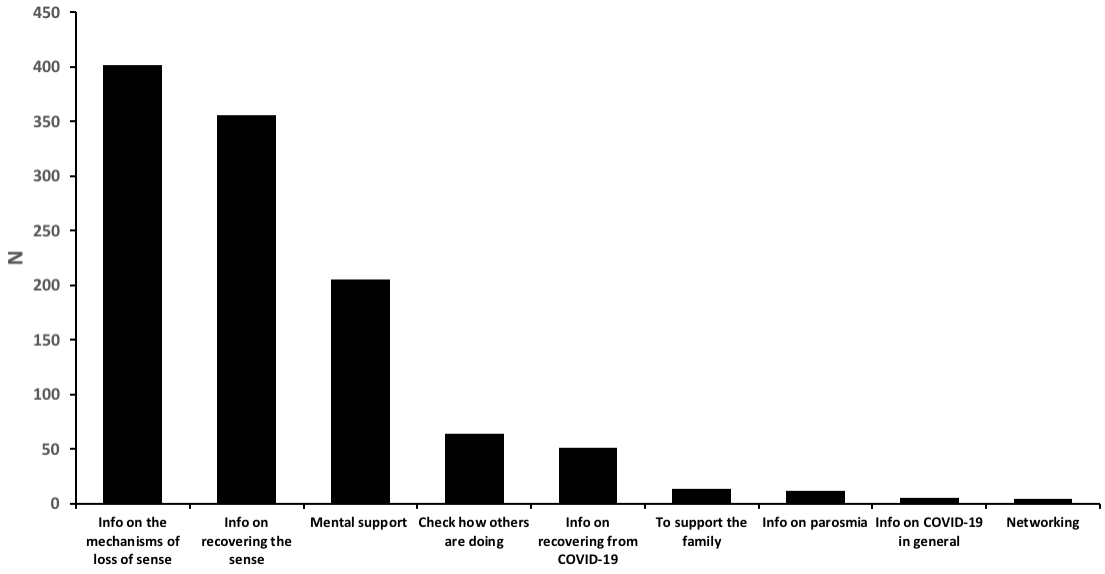
Reasons to join the membership group. Multiple selections allowed and adding options was allowed.

The self-isolation situation, staying at home, itself can cause anxiety. With symptoms of suspected COVID-19 and without a COVID-19 test and medical treatment, it is not hard to imagine that this situation causes high anxiety. It is possible during self isolation to obtain information from the internet and, if available, to join a patient group to exchange information. We asked the members the reasons that they joined the group. We found that the top three reasons were *to obtain the information about the mechanisms of sense loss* (n=401), *information on how to recover the senses* (n=355), and *mental support* (n=205), which accounted for 86.65% (n=961) of the 1109 responses (Figure 5). The sum of the responses related to information (5 out of 9 options; n=823, 74.21%) and mental support of themselves or their family (2 out of 9 options; n=218, 19.66%) accounted for over 93% of the reasons to join the group. If we consider that the two other options, *networking* and *checking how others are doing*, are relatively similar to mental support, it is possible to say that obtaining information and mental support are the two major reasons for joining the group.

**Figure 6:**
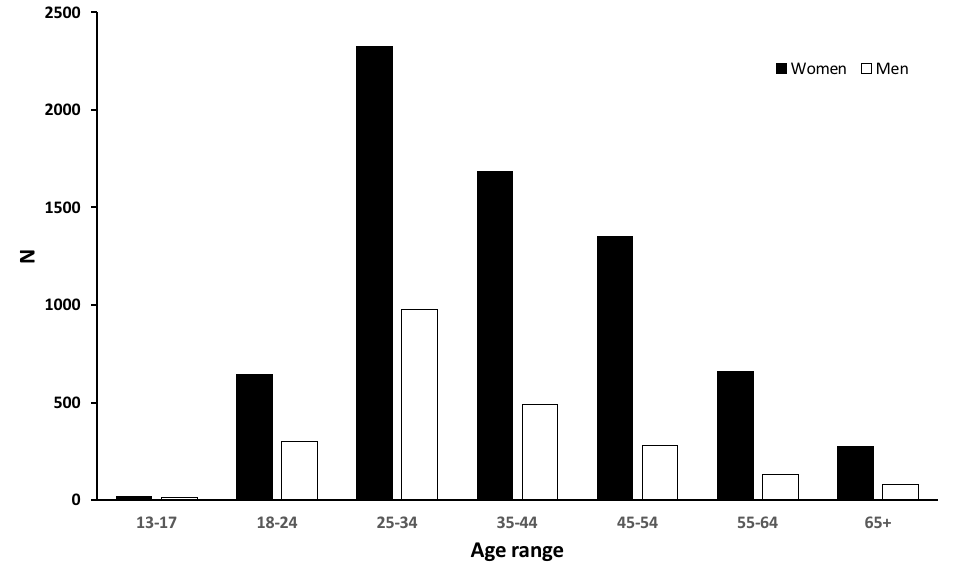
Number of members of the COVID-19 Smell and Taste Loss group sorted by age and sex.

Demographic data for the registered members (n=9227 as of October 12, 2020) of the social media site is shown in Figure 6. The number of women in the group (n=6995, 75.38%) was about three times more than men in the group (n=2272, 24.62%). This number included the members who were administrators of the group or scientists, and not patients, although the estimated percentage of these nonpatient members in the group was minimal. There was a peak in the number of members in the age range between 25-34 years in both men and women (Figure 6). This high number in the rather young ages could be due to the use of social media, although there are multiple other reports on patients with COVID-19 with anosmia and ageusia, which are not using social media, that show the same tendency [6]. On the other hand, in other membership groups of AbScent on social media, the average age is higher, which also suggests that this is specific to COVID-19 and not due to the use of social media. We do not know yet if this is due to the tendency that older-aged people develop severe conditions when they contract COVID-19 and are hospitalized or reach a severe condition such that they are unable to use the internet, or if the symptoms of the senses are specific to patients of younger ages.

**Figure 7:**
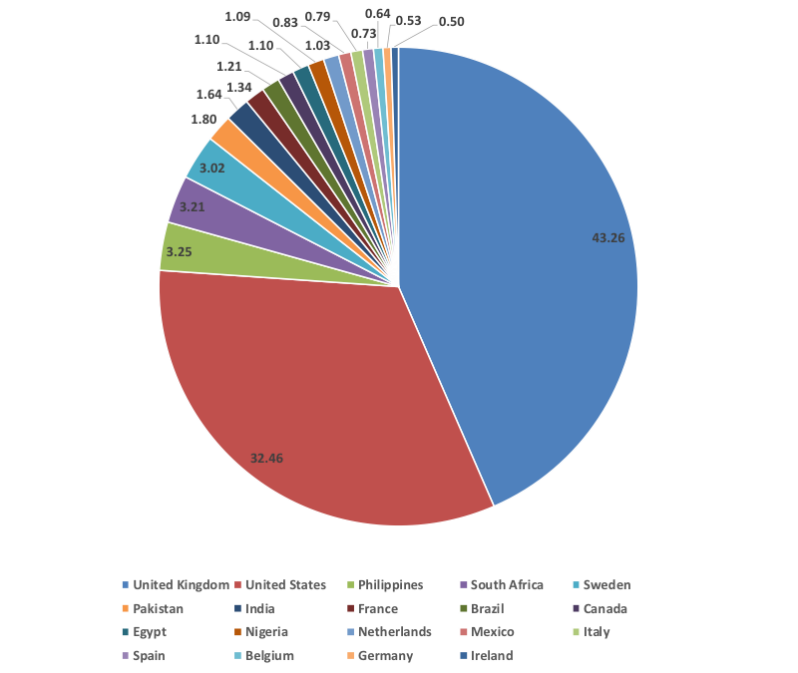
Top 20 countries with a large number of members. Total number of members in these 20 countries was 8654 as of October 12, 2020. The numbers shown inside or outside of the figure indicate the percentage of members of each country.

Figure 7 shows the top 20 countries of the members as of October 12, 2020. Where the patients were located may have had some influence on the symptoms, considering the mutation of the reported virus [21,22]. There were a total of 99 countries that had members in the group. The fact that it was possible to join the group from anywhere the internet was available, if there was no federal restriction on joining a social media group, was one of the benefits for suspected patients staying at home without medical treatments. The top 2 countries, however, were the United Kingdom and the United States, comprising 75.7% (United Kingdom: n=3744; United States: n=2809; total of the 20 countries: n=8654 members) of the number of members of the top 20 countries. This suggests that it is easy for the people in the countries where English is used to join the social media group. This may explain the names of the third and fourth top countries as well. The Philippines and South Africa are the third and fourth countries, respectively, in the number of members, and English is one of the official languages in these countries. There were rather few members from the countries in Europe even though the pandemic was severe there (eg, Italy: n=68, 0.79%), which could be due to the language barrier.

### Symptoms

In December 2019, when the first reports on the outbreak started in Wuhan, China, the major symptoms reported were shortness of breath, cough, fever, and diarrhea. However, it has become clear that many patients who contracted the virus are asymptomatic yet contagious [3-5]. In addition, the symptoms of the symptomatic patients are more varied than first reported (Figure 2). Many patients reported symptoms that suggested encephalitis (headaches, foggy brain, memory loss, hallucinations, pain in the upper end of the nose suggesting inflammation in the olfactory bulb, ear pain, and pain in the back of eyes; Table 1). In addition, some patients also reported a skin rash and tingling in the legs (Table 1), which also has been reported by other recent publications [23].

**Table 1:** Symptoms other than anosmia or ageusia reported by COVID-19–induced anosmia or ageusia patients.

Region and symptoms			Patients, n
**Head**			
	Headache, migraine, pressure in head	143	
	Dizziness, light head, vertigo	25	
	Depression, anxiety	22	
	Foggy brain	8	
	Insomnia	7	
	Forgetful	2	
	Hallucination	1	
	Disoriented	1	
	Nightmare	1	
**Nose**			
	Congested sinus, pressure in sinus, stuffy nose	67	
	Burning nose, nose pain, inflammation in nose, nasal pressure	51	
	Running nose	21	
	Sneeze	11	
	Bloody nose	8	
	Dry nose	4	
	Dried glue mucus	2	
**Respiratory system**			
	Cough	91	
	Sore throat, burning sensation in throat	47	
	Chest pain, chest tightness, burning lungs	43	
	Short breath, difficulties in breathing	31	
	Congested throat	4	
	Wheezing, harsh voice	3	
	Throat congestion	1	
**Eyes**			
	Eye pain, sore eyes	18	
	Dry eye	3	
	Light sensitivity	2	
	Pink eye, red eye	2	
	Watery eye	2	
	Vision affected, difficulties in distance vision	1	
**Mouth**			
	Dry mouth, sore mouth	6	
	Tooth pain, tooth sensitivity	3	
	Tingling in tongue, sensation in tongue	2	
	Film on tongue, thrush	2	
	Dry lips	1	
	Burning mouth	1	
**Ear**			
	Ear pain, ringing ears, tinnitus	20	
	Muffled ear, ear congestion, ear infection	4	
	Deafness in ear	1	
**Gastrointestinal system**			
	Nausea, upset stomach	17	
	Diarrhea, off stomach	14	
	No appetite	9	
	Stomachache	1	
**Other**			
	Fatigue, drained, winded	117	
	Fever, feverish	81	
	Body ache include all parts other than in the head area, muscle ache	74	
	Chills, shiver, chills and sweating	19	
	Rash on body	8	
	Malaise	6	
	Pins and needles in leg	6	
	Sensitive skin, sore skin pain	3	
	Burning up	2	
	Numb at toe, numb at hands	2	
	Face hurt, pressure in face	4	
	Facial palsy	2	
	Skin wrinkle	1	
	Dry skin	1	
	High blood pressure	1	

During the time period that the posts from the members were recorded (March to the end of June 2020), there were 355 members who posted reports of their smell or taste dysfunction and other symptoms they had. Of these 355 members, the percentage of patients without other symptoms comprised 14.93% (n=53), and 85.07% (n=302) of them had other symptoms than the loss of their senses. Almost 10% had recovered their senses by the time this report was summarized (recovered vs not recovered: n=34, 9.58% and n=321, 90.42%). The percentage of patients who recovered their senses was higher in the patients who did not have other symptoms besides anosmia and ageusia (recovered with other symptoms vs without other symptoms: 25/302, 8.28% and 9/53, 16.98%).

There were 123 members who reported that they had other symptoms and reported the timing of the onset of the loss of senses and other symptoms. Of the 123 members, 112 (91.06%) members reported that other symptoms started first. The average difference of the days between the onset of other symptoms and the onset of the loss of senses was 4.2 days. There were 6 (4.9%) members who reported that the onset of the loss of senses occurred on the day other symptoms started, and 5 (4.07%) members who reported that the loss of senses occurred before other symptoms started. The average difference of the days between the onset of the loss of senses and other symptoms was 1.6 days when the loss of senses preceded the onset of other symptoms.

**Figure 8:**
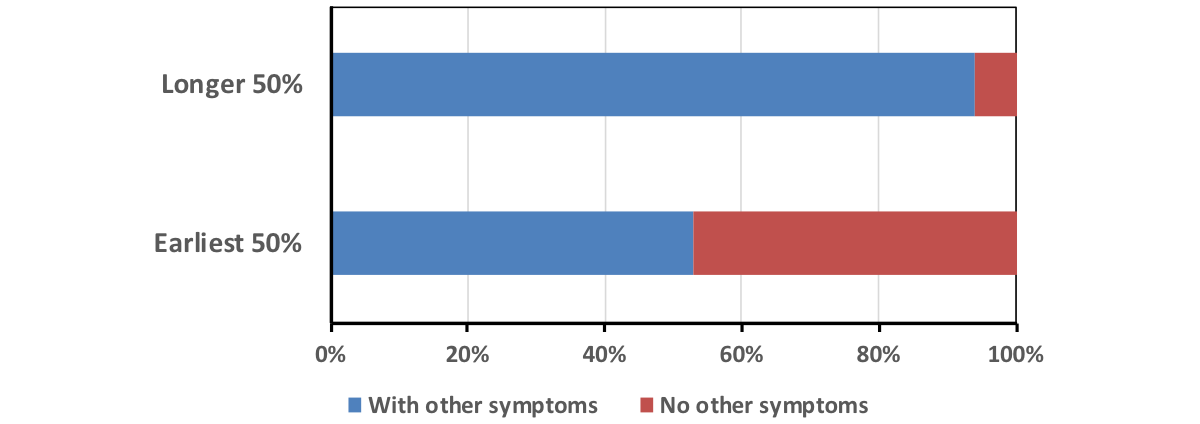
Recovery of senses. Earliest 50%: patients who lost their senses and recovered; longer 50%: patients who lost their senses and took a longer time to recover.

The duration of time until recovery varied largely, with some people regaining their senses within a week and some people who lost their senses at the beginning of the pandemic and still had not recovered their senses at the end of June 2020. As we found the tendency that patients without other symptoms had a rather higher recovery rate, we examined if these differences correlated with the duration to recover their senses as well. Table 2 shows the 34 patients who recovered their senses in the order of the days until recovery. When the 34 patients were divided into the top 50% and the bottom 50% (fast recovery group and slow recovery group; n=17 for both), the average days to recover was 12.9 days for the fast recovery group and 41.3 days for the slow recovery group. In the fast recovery group (n=17), there were 8 (61.54%) patients without other symptoms, whereas in the slow recovery group (n=17), there was only 1 (7.69%) patient who did not have other symptoms, and there was a statistically significant difference in the duration to recovery (Fisher exact test was used: *P*=.02; Figure 8). These results indicate that patients without other symptoms than the loss of the senses had the tendency to recover earlier. This, however, did not indicate that patients without other symptoms will always recover their senses faster. Of the 321 patients who reported they had not recovered their senses by the time we summarized this report, there were 44 (13.71%) patients who reported they did not have other symptoms. This suggests that there may be several causations in the loss of senses (ie, one of these could be the severity of the overall COVID-19–related symptoms and some other factors could also be involved, affecting the recovery process).

Patients with anosmia and ageusia occasionally reported distorted smell and taste (parosmia and dysgeusia) as well as the smell and taste without the sources of the smell or taste (phantosmia and phantogeusia). Table 3 summarizes the reports from the patients. Although there are various ways to describe the nature of phantosmia and phantogeusia, a smell and taste of smoke and burnt material was most frequently reported.

**Table 2:** Days until the recovery of senses sorted by the days and the symptoms.

Order	Until recovery of senses (days)	Symptoms other than anosmia and ageusia
1	5	Low grade fever, fatigue, insomnia
2	9	No other symptoms
3	9	No other symptoms
4	11	Fatigue, chest pain, short breath, no fever, no cough
5	12	No other symptoms
6	13	No other symptoms
7	13	No other symptoms
8	14	Cough, nausea
9	14	No other symptoms
10	14	Slight fever, runny nose
11	14	Fever, aches, chills, sore throat, headaches, rash
12	14	No other symptoms
13	14	No other symptoms
14	15	Nose pain, fatigue, headaches
15	15	Fatigue
16	17	Cold, runny nose, fatigue, sore eyes, congestion
17	17	Fatigue, cough
18	21	Burning nose, fatigue, eye pain, shiver
19	21	Fatigue, sore throat
20	23	Fever
21	27	Dizziness, disorientation, foggy brain, insomnia, ear congestion
22	28	No other symptoms
23	30	Back pain, sore throat
24	31	Burning nose, headache, fatigue, sinus infection
25	32	Nose is super dry and inflammation is high
26	35	Backache, low fever, very mild nasal congestion and mild difficulty breathing.
27	38	No fever, chills, coughs, headache, muscle ache, fatigue
28	42	Fatigue, feverish, cough
29	42	unwell, sore throat, breathless, headaches, fatigue, nausea, tinnitus
30	47	Fever, headache, fatigue, sore skin
31	56	Cough, fever, muscle ache, lethargy
32	69	Fatigue, headaches, fizzy, burning nose
33	77	Fatigue, headaches
34	83	Fatigue, headache, nasal pain

**Table 3:** Types of phantosmia and phantogeusia.

Types of phantosmia and parosmia	Reported cases, n
Smoke, ashtray, cigarette, burnt, fire, dusty	43
Chemical	7
Ammonia, vinegar	6
Metallic	5
Garbage, rotten	3
Skunk	1
Other expressions: weird, strange, distorted	6

### Case Studies

Here, we show several examples of reports from the patients. In selecting the examples, we first classified the 355 members who posted reports of their smell or taste dysfunction during March and June 2020 into the patients who recovered (fast recovery group and slow recovery group) and had not recovered, and those with or without other symptoms than anosmia and ageusia.

Case 1 represents a fast recovery group patient who recovered early and did not have other symptoms. The length of time until recovery of smell and taste was 9 days. This patient was not tested for COVID-19. The dates shown in Textbox 1 were added when they were recorded in Excel.

**Case 1: fast recovery group without other symptoms (original posts).**

**April 7, 2020 (Tuesday)**

- “Woke up and Lost all sense of smell on Thursday. Can’t taste can only taste salt or sweet. Smell seems to be coming back it’s now at about 10% maybe lower because I can only smell garlic and onion powder. Can’t smell perfume at all. I am day 6. I am praying it’s returning. Brother in law lost his smell for 11 days and it’s returned fully”

**April 8, 2020 (Wednesday)**

- “I woke this morning and I can smell my toast in the toaster and also zoflora cleaning product can also taste the toast as well I would say it’s come back 30% so happy it’s improving daily thank god.”
- “Quick update lost all sense of smell last Thursday complete loss no taste or smell. Yesterday i could smell spices on close inspection. This morning I could smell my toast in the toaster and also taste it it’s still not 100% but I would say it’s now at about 30% smell 50% taste it is getting better each day I’m now on day 7 since losing it so the general time frames are generally 7-14 days but if yours isn’t then don’t panic it will come back in time.”
- “It came back together once my smell returned yesterday taste returned but can taste more than I can smell it’s 40% smell 60% taste which am hoping will improve daily I’m On day 7 too.”

**April 9, 2020 (Thursday)**

- “Hi all update to help anyone currently feeling scared and alone try and not to panic it will come back mine is now back 75% I’m day 7 I cried tonight when I ate dinner because I could taste and smell it very scary when all sense goes but the joy when it returns we often take this sense for granted please don’t worry it will return”

**April 11, 2020 (Saturday)**

- “100% back”

Here is another example from the fast recovery group, but the patient had other symptoms. The patient reported recovery of the senses in 13 days (however, at the time point of summarizing this report, we have heard that this patient started to experience parosmia starting from about 2 months after the recovery of the senses). The dates shown in Textbox 2 were added when they were recorded in Excel.

**Case 2: fast recovery group with other symptoms (original posts).**

**March 28, 2020 (Saturday)**

- “I’ve noticed this other weird phenomenon and wanted to see if you all were experiencing it too. Since I got my first possible CV19 symptom 13 days ago (slight cough), whenever I shower my skin on my hands wrinkle up super fast...like within 5 minutes of being in the shower. It usually take a good 30 minutes for that to happen to me. Now I barely shampoo and my hands are all wrinkled. My wife noticed it happening to her too although she has had no symptoms. Anyone else notice this weird thing? Ps...my smell is coming back! It’s about 40-50% today and I’m on day 7. I hope it continues!!!”

**March 29, 2020 (Sunday)**

- “I’m on day 8 and yesterday was the weirdest day. I had the strange sense of a smell whenever I inhaled, like you, but mine was like a burning sour smell. And I got a slight metallic taste when I was eating food. Not going to lie, it was pretty awful. In fact, at the end of the day I started feeling sick to my stomach with the feeling that I had been smelling sour milk for hours and hours. Luckily today, that’s completely gone. Now I’m back to smelling things about 50%. I think it’s called Phantosmie. I don’t know, I’m going to do more research today.”

**April 3, 2020 (Friday)**

- “I just wanted to write a positive post for those who are feeling anxious and scared. I’ve got my smell and taste back! It is GLORIOUS. Everything is so flavorful and the smells are incredible. I lost both senses 13 days ago and had absolutely no improvement for 5-6 days. I was so scared that it might never come back and it was very depressing. On day 5 or 6 I could just get a wiff of a few things that I passed by maybe at about 5%. It stayed there for a couple days and then slowly started to get better. I personally had a lot of ups and downs where one day it might be at 60% and then the next day back at 30%. I was feeling rather frustrated because I couldn’t tell if I was really getting better or not. Well I’m happy to say that the last two days I’ve been able to taste everything I’ve eaten and smelled things I haven’t smelled in almost two weeks!! I know it might drop again before being at 100% consistently but I feel hope that I will fully heal for the long haul. Those of you who are where I was a week or so ago...don’t give up. Try to stay hopeful even though it’s hard. I want this to encourage anyone who feels nervous right now. It’s a possibly that things will be ok.
  “PS...I’m pretty sure I had CV19 but couldn’t get tested because I got don’t fit the criteria.”
- “I had a dry cough and then a full head cold and I lost my senses once my cold started to get better. I did have one day of burning nose and chemical taste right before I started really getting better with my smell and taste.”

The next example is a patient of the slow recovery group who recovered after 42 days and had other symptoms. The dates shown in Textbox 3 were added when they were recorded in Excel.

**Case 3: slow recovery group with other symptoms (original posts).**

**April 8, 2020 (Wednesday)**

- “I started getting covid symptom April 1st and on day 4 my sense of smell and taste suddenly 100% dissapeared. Now this was only 4 days ago but it already feels like a lifetime...I can't even remember what it's like to taste food. I'm just wondering, I've seen that this could be caused by a zinc deficiency? Coincidently I had just started taking zinc when the covid symptoms started so I was taking it, but now that I've actually started researching it apperently a certain form of zinc (I believe it was a nasal spray) had caused permenant loss of smell for many people so now I'm kindof hesitant to even take the pills...So confusing!!!”

**May 16, 2020 (Saturday)**

- “just wanted to share my story before I leave this group to provide hope to people. I got COVID symptoms April 1st (weakness, fatigue, feverish, dry cough) and woke up April 4th with zero taste or smell. I was never actually tested. I had 5 days with absolutely nothing (which was absolutely horrible, I can't even imagine people who have to deal with that for more than a month it was literally all I thought about) then I was finally able to get a tiny whiff of one of my essential oils (lemon grass haha) which gave me hope that it was not permenant. It was soooo slow coming back, like I would say it only improved max 2% a day. It is only yesterday that I realized I think I am finally at 100% back to normal, so I would estimate it took a month to slowly come back. Throughout that month I would wake up almost every day with that phantom “chemically plasticy burning” smell/sensation which gradually reduced in strength, I do remember smelling it only a few days ago. Now I guess my recovery is sort of on the fast side, which I would attribute to my healthy lifestyle. I generally eat a plant focused diet, with minimal meat, dairy and processed foods. Since I was sick I have had barely any alcohol and obviously no smoking. In the beginning I was taking vitamins daily such as vit C, D, zinc and oil of oregano. I also tried to incorporate many anti-inflammatory foods into my diet such as tumeric, garlic, ginger. Who knows how much this actually helped but I'm sure it didn't hinder.”

The last example is of a patient who has not recovered their senses yet. The dates shown in Textbox 4 were added when they were recorded in Excel.

**Case 4: not recovered, with other symptoms.**

**March 29, 2020 (Sunday)**

- “March 13th - sudden and complete loss smell and taste. Before that date I have been suffering some headaches. March 16 to 18 – fatigue and dizziness. Now I feel ok physically but smell and taste is still gone. After 12-13 days later I was able to smell coffee grains but faintly. My testers are coffee, vicks, perfume for sniffing. I’m about 10% and it comes and goes back. Taste was completely gone I could not even identify salty, spicy etc. Now I can identify them but flavor is missing. I feel 15-20% improvement but not so sure.”

**April 2, 2020 (Thursday)**

- “I could smell Vicks around day 15-16. Now in day 20. Coffee, garlic, mint etc. are OK but rest is not good”

**April 14, 2020 (Tuesday)**

- “Day 32 still weak. It comes and goes even during the day. Early mornings and evenings are worse. Still need to get close to be able to smell and taste is way behind.”

**April 24, 2020 (Friday)**

- “Hi all! It has been around 10 weeks for me and I cannot even say the percentage :) How do you define it? For example, I feel 100% for mint, coffee or other strong things but 0% for basil interestingly - once upon a time it was my fav :D Besides, all bad smells are totally gone. Sometimes I can smell strong smells in the air - if there is garlic in the food for example. And as many others I'm experiencing fluctuations hour by hour and day by day. Still feel improving but sometimes I feel quite desperate and it's really diffucult to stay always postive! Good luck to all of us!”

## Discussion

### Benefits and Limits of Social Media

The year 2020 has become a tragic year with the pandemic that has over 67 million cases and over 1.54 million deaths worldwide as of December 6, 2020. At the early stage of the pandemic, many suspected COVID-19 patients could not get tested because of the overwhelming number of patients with severe conditions at the hospitals. This suggests that the actual number of patients with COVID-19 could be far more than the officially reported number. Many of the suspected COVID-19 patients with mild to moderate symptoms self-isolated themselves without medical treatment. These suspected COVID-19 patients had to depend on the internet to obtain information, and many of them joined membership groups on social media, which were founded to support patients with COVID-19.

In this study, we analyzed the information in the social media database of suspected COVID-19 patients, with particular attention to their olfactory and gustatory dysfunction. About 70% of them could not get tested at all or could not get tested immediately and received the antibody tests later. Furthermore, about 90% of them either stayed at home with generic medicines or supplements, or stayed at home without any medicines. These results suggest the limited access of the patients with mild to moderate symptoms to medical services. In the aspect of olfactory and gustatory dysfunction, it was demonstrated that patients who were asymptomatic other than the senses comprised about 15% of patients. Recovery of the senses was higher in the patients who were asymptomatic besides having anosmia and ageusia. Most patients experienced other symptoms first and then lost their senses, on average, 4.2 days later. Patients without other symptoms tended to recover earlier (*P*=.02). Patients with anosmia and ageusia occasionally reported distorted smell and taste (parosmia and dysgeusia) and experiencing or perceiving the smell and taste without the sources of the smell or taste (phantosmia and phantogeusia). Olfactory dysfunction of a large portion of patients with COVID-19 persisted at least several weeks to months.

Through the analyses of the reports, we have noticed the benefits and limits of using social media (Textbox 5). There are many benefits for patients, which suggest that these membership groups can be a beneficial system for patients to provide information and mental support. For researchers, there are some limits that require improvements to use social media membership groups to enhance understanding of the COVID-19 symptoms and conditions of the patients. These limits are due to the free nature of the involvement in the activities of the group, which causes difficulties in following up with each patient, and control of the conditions. The reports are based on each patient’s subjective experience that the same symptom at similar severity could be experienced as a difficult experience by one person and as an easy experience by another. Some modifications in the way of organizing these membership groups may be able to reduce these limits for the researchers in the future. Demographic data revealed that there were about three times more women than men, and the range of age showed a peak in the age group of 25-34 years. The number of members in each age group decreased in the groups of higher age. These results on the demographic trends may reflect the trends that the symptoms of the younger people are milder compared to older people, and thus, they stayed home without medical treatments. However, there are also possibilities that women used social media more than men and that younger people used social media more than older people to obtain information on COVID-19. There were, in fact, a few cases that wives who joined the group instead of the husbands with suspected COVID-19 and mothers who joined the group instead of their small children who were suspected COVID-19 patients. These are the possible aspects of the social site structures that can skew the information.

**Benefits and limits of using social media.**

**Benefits**

- (Patient) Possible to post *immediately* without waiting, compared to doctor visits, which usually require appointments
- (Patient) Patients who have mild/moderate symptoms can obtain and exchange *information*
- (Patient) Patients who have mild/moderate level of symptoms can *mentally support* each other
- (Patient) Possible to join from anywhere in the world if there is access to internet
- (Researcher) If the number of members increase, it is possible to obtain large numbers of data
- (Researcher) Possible to understand the patients’ conditions, emotional responses, their experiences with their doctors from their posts

**Limits**

- (Patient) Possibility to join is limited to the areas where there is access to internet and to countries without federal restrictions to join internet groups
- (Researcher) There is no control group
- (Researcher) Some people are tested for COVID-19 and some are not, and we do not know which person is tested by the reports unless the person writes this each time
- (Researcher) Cannot get the same people to respond to each poll
- (Researcher) Everything is based on self-reporting and it is difficult to get precise information on the symptoms to diagnose
- (Researcher) Members often leave without notification and it is difficult to follow up

### Symptoms

Among the symptoms, we especially focused on smell and taste dysfunction, which is now known as one of the major biomarkers of COVID-19. The use of social media allowed members to join the group from around the world. Although there is a limit in the range of information that can be obtained through social media (eg, accurate diagnosis of the patients), the use of it enabled us to obtain the symptoms and the progression of these symptoms from the patients who did not or could not visit the hospitals.

The current analysis demonstrated that about 90% of the 355 patients in the *COVID-19 Smell and Taste Loss* group that reported their subjective smell or taste disturbance did not recover, at least completely, for months. These results are in sharp contrast to the previously reported findings that 80% of smell and taste dysfunction in patients with COVID-19 is recovered within a few weeks [9,24-26]. This discrepancy may be due to several reasons: the differences in the patient populations (ie, the patients in the previous studies were mostly in-patients whereas over 99% of the patients of this report had never been hospitalized and most of them did not or could not have access to medical treatments at a hospital), this is a self-selected group who joined because of their smell loss, and one limit of social media, which is the inability to confirm the recovery unless the patients post their recovery to the group.

The pathophysiology of human olfactory dysfunction is divided into three major categories. One is an airflow problem, where the airflow toward the olfactory cleft is blocked by mucosal swelling or mucus hypersecretion [27-29]. The second is a sensorineural problem, where the perception of odorants is disturbed due to damage to the olfactory mucosa, such as olfactory neural degeneration, downregulation of the odorant molecules on the olfactory neurons, or impaired transport of the odorant molecules to the olfactory receptors due to secretory dysfunction of Bowman glands [27-29]. The third is a central nervous problem, in which the olfactory pathway from the olfactory bulb to the olfactory cortex is damaged [27-29]. Airflow problems could be resolved in a relatively short time as mucosal swelling by viral infection decreases in several days to weeks, and usually the smell problem could fluctuate in its severity during the time course, depending on the nasal condition. In contrast, sensorineural and central nervous problems are usually severe, do not fluctuate, and take a longer time (months to years) for recovery. Postviral olfactory disorder, an olfactory problem developing after an upper respiratory viral infection, is usually considered as a combination of a sensorineural and central nervous system problem [27-29]. In contrast to such current concepts, previous studies reported that a large portion of patients with COVID-19 recovered from their olfactory dysfunction within a few weeks [9,24-26], suggesting that the problem was due to an airflow problem. In fact, a case report of olfactory dysfunction in a patient with COVID-19 showed mucosal swelling in a restricted area of the olfactory cleft on magnetic resonance images [30]. However, our analysis, as well as some more recent studies [31], demonstrated that a larger number of those with olfactory dysfunction were not resolved in such short time frames, suggesting that a neural problem could be also involved in COVID-19–induced olfactory dysfunction. Previous reports suggest that the virus may migrate along the peripheral and central nervous system. Studies using animal models have shown that human coronaviruses can make axonal transports enabling neuron-to-neuron propagation [32]. Development of parosmia, a sign of neural olfactory dysfunction, supports this hypothesis.

Our analysis also demonstrated that the smell problem of patients with COVID-19 tends to recover sooner when the patients only have smell dysfunction, not multiple COVID-19 symptoms. The reason for this finding is unclear, but one possible explanation is that, for the outpatients without access to medical treatments, recovery from olfactory dysfunction is faster if the patients have a lower viral load and therefore fewer symptoms. In other words, olfactory dysfunction may be one of the most sensitive indicators of SARS-CoV-2 infection. In fact, the percentage of patients showing anosmia or ageusia was especially high in the asymptomatic to mild level patients [9]. This indicates that anosmia and ageusia are several of the important symptoms to diagnose COVID-19 in the early stages, and to detect patients who are asymptomatic or have mild symptoms [12,15]. The official classification of the levels of illness is based on the ability to breathe, chest pain, and ability to talk or move [1], although often the ability to breathe becomes the major criteria. The level called *mild* refers to the lack of these signs of severity, although often the pains and severity of symptoms in patients classified to have mild or moderate symptoms are not mild or moderate (Table 1). Some patients with mild symptoms have fatigue and pain in various parts of the body for a prolonged length of time [33]. This indicates that patients with COVID-19 need to be diagnosed as soon as possible to enable immediate treatment not only to avoid the symptoms turning into a severe condition but also to avoid the prolonged time of these symptoms.

As described in this study, social media can become an important platform to collect data from outpatients, especially in the era of infectious disease pandemics, when hospitals are overwhelmed with patients that have severe conditions and patients with mild or moderate symptoms are often overlooked. Following the outbreak of COVID-19, virtual visits to doctors have started to become more popular, which can provide medical service without in-person visits. The benefits of social media are that patients can post their symptoms and questions whenever they are in need without appointments and can receive responses from the public, either patients, nurses, or doctors, through the internet, which enables real-time monitoring of the infected condition, patient symptoms, and the clinical course. In the United States, there is a project called DETECT, which uses a smartphone and a wristband to sense changes in the heart rate, which can be an indicator of changes in health conditions [34]. This device also allows the movement of people to be tracked, providing epidemiologically useful data. However, DETECT cannot distinguish between changes due to COVID-19 or other causes. The use of social media in addition to these other media (ie, virtual doctor visits and portable tracking devices) can become a strong monitoring method in the era of infectious disease pandemics. What is necessary to further strengthen the merits of social media could be the establishment of a system that serves as a route from the social media to, for example, virtual doctors or clinics nearby. At the current stage, obtaining enough information to provide diagnosis and treatments through social media sites contains challenging aspects for medical professionals. A system that guides patients from social media to the adequate medical support they need, to counselling, to other types of support systems, and the establishment of the rules in detail to organize such systems will help social media to become one of the new tools for the medical system in the near future.

### Principal Results

Our analysis of a social media database of COVID-19 patients’ posts demonstrated that olfactory dysfunction of a large portion of patients with COVID-19 persisted at least several weeks to months. This information is important in understanding the pathophysiology of COVID-19–induced anosmia. Although accurate diagnosis of patients is not always obtained with social media–based analysis, it may be a useful tool to collect a large amount of data on symptoms and the clinical course of worldwide, rapidly growing, infectious diseases.

### Limitations

Our study explores the benefits and limits of using social media membership groups in studying the symptoms of COVID-19. The patients are suspected COVID-19 patients and not all of them have been tested for COVID-19, but all have experienced loss of the senses of smell and taste.

### Comparison With Prior Work

This is the first study to determine the benefits and limits of using social media in studying the symptoms of COVID-19.

### Conclusions

Social media membership groups can function as a source of information and mental support for patients with COVID-19. Although some improvements in the system are necessary for its use for research purposes, it is a rare source of a large amount of data for researchers as well.

## Abbreviations

CDC: Centers for Disease Control and Prevention
WHO: World Health Organization
